# Zika Virus Infection Induces Elevation of Tissue Factor Production and Apoptosis on Human Umbilical Vein Endothelial Cells

**DOI:** 10.3389/fmicb.2019.00817

**Published:** 2019-04-24

**Authors:** Fatih Anfasa, Marco Goeijenbier, Widagdo Widagdo, Jurre Y. Siegers, Noreen Mumtaz, Nisreen Okba, Debby van Riel, Barry Rockx, Marion P. G. Koopmans, Joost C. M. Meijers, Byron E. E. Martina

**Affiliations:** ^1^Department of Viroscience, Erasmus University Medical Center, Rotterdam, Netherlands; ^2^Faculty of Medicine, Universitas Indonesia, Jakarta, Indonesia; ^3^Department of Internal Medicine, Erasmus University Medical Center, Rotterdam, Netherlands; ^4^Department of Plasma Proteins, Sanquin Research, Amsterdam, Netherlands; ^5^Department of Experimental Vascular Medicine, Academic Medical Center, University of Amsterdam, Amsterdam, Netherlands; ^6^Artemis One Health Research Institute, Delft, Netherlands

**Keywords:** Zika virus, endothelial cells, HUVECs, tissue factor, secondary hemostasis, apoptosis

## Abstract

Zika virus (ZIKV) infection is typically characterized by a mild disease presenting with fever, maculopapular rash, headache, fatigue, myalgia, and arthralgia. A recent animal study found that ZIKV-infected pregnant *Ifnar*^−/−^mice developed vascular damage in the placenta and reduced amount of fetal capillaries. Moreover, ZIKV infection causes segmental thrombosis in the umbilical cord of pregnant rhesus macaques. Furthermore, several case reports suggest that ZIKV infection cause coagulation disorders. These results suggest that ZIKV could cause an alteration in the host hemostatic response, however, the mechanism has not been investigated thus far. This paper aims to determine whether ZIKV infection on HUVECs induces apoptosis and elevation of tissue factor (TF) that leads to activation of secondary hemostasis. We infected HUVECs with two ZIKV strains and performed virus titration, immunostaining, and flow cytometry to confirm and quantify infection. We measured TF concentrations with flow cytometry and performed thrombin generation test (TGT) as a functional assay to assess secondary hemostasis. Furthermore, we determined the amount of cell death using flow cytometry. We also performed enzyme-linked immunosorbent assay (ELISA) to determine interleukin (IL)-6 and IL-8 production and conducted blocking experiments to associate these cytokines with TF expression. Both ZIKV strains infected and replicated to high titers in HUVECs. We found that infection induced elevation of TF expressions. We also showed that increased TF expression led to shortened TGT time. Moreover, the data revealed that infection induced apoptosis. In addition, there was a significant increase of IL-6 and IL-8 production in infected cells. Here we provide *in vitro* evidence that infection of HUVECs with ZIKV induces apoptosis and elevation of TF expression that leads to activation of secondary hemostasis.

## Introduction

Zika virus (ZIKV) is a re-emerging arbovirus that belongs to the genus *Flavivirus* of the family Flaviviridae. ZIKV was first isolated from a rhesus macaque in 1947 in Uganda and has not been recognized as an important viral pathogen until the recent outbreaks in the Americas. Infection is asymptomatic in the majority of cases (80%) and symptomatic patients develop a febrile illness similar to many other infectious diseases (Baud et al., 2017). Clinical symptoms are usually mild and last for 3–7 days without further complications. However, during the recent outbreaks, severe symptoms and complications were reported, including Guillain-Barré syndrome, (Parra et al., 2016) severe birth defects, (Brasil et al., 2016b) fetal death (Sarmiento-Ospina et al., 2016; Sharp et al., 2016; Zonneveld et al., 2016), and coagulation disorders (Wu et al., 2017). The mechanism of these complications, especially coagulation disorders, are still not fully understood.

Under physiological conditions, hemostasis is orchestrated by the coagulation and the fibrinolytic systems. Endothelial cells (ECs) play an important role in regulating the activities of pro-and anti-coagulation and fibrinolysis through expression and production of several important mediators, including tissue factor (TF), tissue factor pathway inhibitor (TFPI), tissue plasminogen activator (tPA), plasminogen activator inhibitor type-1 (PAI-1), and thrombomodulin (Levi et al., 2003). TF is an important factor that initiates the activation of secondary hemostasis. Several factors have been shown to activate and down-regulate this protein (Levi and Poll, 2015; Bester and Pretorius, 2016). For instance, interleukin (IL)-6 and 8 are pro-inflammatory cytokines that regulate TF expression on several cells such as human umbilical vein endothelial cells (HUVECs) and monocytes (Wada et al., 1995; Neumann et al., 1997). In addition, apoptotic cells could also activate the coagulation system by increasing the surface TF expression (Greeno et al., 1996). It has been shown that several viruses activate the coagulation system especially through TF (Ruf, 2004). For instance, treatment of Ebola virus (EBOV) infection with a recombinant inhibitor of factor VIIa/TF was shown to result in prolonged survival, which was associated with reduced activation of coagulation and fibrinolysis (Geisbert et al., 2003). Dengue virus (DENV), another flavivirus, has been shown to also cause coagulation disorders and ECs have been shown to play a central role in these pathological conditions (Wills et al., 2002; Sosothikul et al., 2007; Basu and Chaturvedi, 2008).

Recently, it was found in a cohort study that 9% of infants from ZIKV-infected pregnant woman were small for their gestational age and the authors speculated that this condition occurred as a consequence of fetal growth restriction or poor placental perfusion (Brasil et al., 2016b). This study led to the hypothesis that coagulation disorder of the umbilical cord could be one of the explanations for abnormal fetal growth due to reduced perfusion which has been shown for cytomegalovirus (CMV) infection (Iwasenko et al., 2011; Lepais et al., 2014). CMV is known to have vascular EC tropism, which causes cell damage and can lead to thrombotic vasculopathy (Rahbar and Soderberg-Naucler, 2005). Recent publications demonstrated that ZIKV also infects ECs *in vitro* (Liu et al., 2016; Tabata et al., 2016; Richard et al., 2017). HUVECs were shown to be more susceptible to ZIKV infection compared to human ECs derived from aorta, coronary artery, and saphenous vein (Liu et al., 2016). Interestingly, a recent report revealed that ZIKV NS1 protein triggers endothelial barrier dysfunction *in vitro* in a tissue-specific manner. The authors found that ZIKV NS1 bind mainly on the surface of HUVECs and brain ECs and cause increased vascular leakage in these primary cells (Puerta-Guardo et al., 2019). *In vivo* evidence also revealed that ZIKV-infected pregnant *Ifnar*^−/−^mice developed vascular damage in the placenta and reduced amount of fetal capillaries (Miner et al., 2016). Furthermore, pregnant rhesus macaques infected with ZIKV developed segmental thrombosis in the umbilical cord (Nguyen et al., 2017). Altogether, these data suggest that hemostatic alterations occur during ZIKV infection. However, there is limited evidence of hemostasis disorder in ZIKV infection. Here, we provide *in vitro* evidence that ZIKV infection of HUVECs induce apoptosis and increased TF production which trigger the activation of secondary hemostasis.

## Materials and Methods

### Cells

Human umbilical vein endothelial cells were harvested from patients as previously described (Goeijenbier et al., 2015). Ethical permission to use the leftover materials from mothers who gave birth at Sophia Children Hospital was obtained from the Erasmus MC medical ethics committee. Only cells up to passage four and from one randomly selected donor were used in this study. The identity of HUVECs was confirmed by flow cytometry using Von Willebrand Factor (vWF) staining. HUVECs were grown in human endothelial-SFM medium (Invitrogen, Life Sciences, United States) containing 20% heat-inactivated fetal bovine serum (HI-FBS, Lonza, Netherlands), 100 U penicillin (Gibco Life Sciences, United States), 100 μg/ml streptomycin (Gibco Life Sciences, United States), 20 ng/ml fibroblast growth factor (Peprotech, United States) and 10 ng/ml endothelial growth factor (Peprotech, United States). Vero cells (ATCC CCL-81, United States) were grown in Dulbecco’s Modified Eagle Medium (DMEM) containing 10% HI-FBS (Lonza, Netherlands), 100 U penicillin, 100 μg/ml streptomycin, 2 mM L-glutamine, 1% HEPES buffer, and 1% sodium bicarbonate (all from Gibco Life Sciences, United States). All cells were grown at 37°C and 5% CO_2_. The cells were tested negative for mycoplasma by PCR.

**Table 1:** Source host, isolation and passage history of the ZIKV strains used in the study.

Lineage	Strain	Source host	Year of isolation	Location	Passage history	Genbank Accession number	EVAg number
Asian	ZIKVNL00013	Human	2016	Suriname	4 × Vero	KU937936	011V-01621
African	Uganda 976	Monkey	1961	Uganda	2 × SMB, 3 × Vero E6, 1 × Vero	NA	007V-EVAg1585

*SMB, suckling mouse brain; Vero, African green monkey kidney cells; Vero E6, African green monkey kidney clone E6 cells; EVAg, European Virus Archive goes Global; NA, Not available.*

### Virus Strains

Two ZIKV strains were used in this study; ZIKV strain Uganda 976 (ZIKV^AF^) was kindly provided by Dr. Misa Korva (University of Ljubljana) (European Virus Archive number 007-EVAg1585) and Zika Suriname ZIKVNL00013 (ZIKV^AS^) was isolated from a female patient in Netherlands who traveled to Suriname (Van Der Eijk et al., 2016). Virus stocks used in this study were prepared on Vero cells. ZIKV Uganda 976 with total passage number (P) 6 and ZIKV^AS^ P4 were used in the experiments. Viral titers were determined on Vero cells by calculating the 50% Tissue Culture Infective Dose (TCID_50_) using the Spearman-Kärber formula (Kärber, 1931) after determining the presence of cytopathic effects (CPE) after 5 days of incubation. As a control, infectious virus was inactivated using beta-propiolactone (BPL) (Sigma-Aldrich, United States, 1:4000 v/v) at 4°C for 24 h. Subsequently, BPL was inactivated by incubating for 1 h at 37°C. All virus stocks were stored at −80°C until use. A summary of isolation history of the ZIKV strains used in this study with related information is provided in Table 1. A phylogenetic tree of the virus strains used in this study was recently published (Anfasa et al., 2017).

### Infection Experiments

Human umbilical vein endothelial cells were seeded into 96-well- (2 × 10^4^ cells per well) (Greiner, United States) or 24-well- (2 × 10^5^ cells per well) (Corning, United States) plates depending on the experiment. After 24 h, confluent monolayers were infected at a multiplicity of infection (MOI) of 0.1, 1, or 5, with infectious, BPL-inactivated virus or a mock (vero cell culture medium) for 1 h at 37°C in 5% CO_2_. Subsequently, the supernatant was discarded and cells were washed three times with RPMI 1640 medium (Gibco, Life Sciences, United States). New medium with 10% HI-FBS was added and cells were cultured for 48 h. All of the experiments were performed according to the local biosecurity safety procedures at the Department of Viroscience, Erasmus Medical Center, which is a WHO collaborating center for hemorrhagic fever viruses.

### Determination of Virus Titer

Virus titers (TCID_50_) in the supernatant were determined by log10 titration of the medium on Vero cells. Presence of CPE was determined at 5 days post-infection (dpi) and the TCID_50_ was calculated using the Spearman-Kärber method (Kärber, 1931).

### Determination of Percentage of Infection

Cells were cultured on 96-well plates and infected with MOI of 0.1 and 1. Infected cells were harvested, then fixed and permeabilized with BD Cytofix/Cytoperm^TM^ solution (BD Biosciences, United States). To minimize background, cells were incubated with 5% normal goat serum (DAKO, Denmark) in BD Perm/Wash solution (BD Biosciences, United States). ZIKV was detected using mouse monoclonal antibody anti-flavivirus group antigen, MAB10216, clone D1-4G2-4-15 (Millipore, Germany) 1:200 dilution and goat anti-mouse IgG conjugated with APC (Life technologies, Netherlands) 1:250 dilution. Non-infected cells and ZIKV-infected cells stained with mouse isotype IgG2a antibody (DAKO, Denmark) were used as negative and isotype controls. The percentage of infected cells was measured using a BD FACS Canto II machine (BD Biosciences, United States). Infected cells were defined as ZIKV Envelope + cells (ENV+) while negative cells were defined as ZIKV ENV – cells (ENV-).

### Immunofluorescence Staining

Cells were grown on 96-well plates and infected with MOI of 0.1 and 1. Cells were fixed with 4% paraformaldehyde (PFA) and permeabilized with a mixture of 0.1% Triton X-100 and ethanol 70% (1:1 dilution). Subsequently, cells were incubated for 1 h with mouse monoclonal antibody anti-flavivirus group antigen, MAB10216, clone D1-4G2-4-15 (Millipore, Germany) 1:200 dilution at room temperature. Next, cells were stained for 1 h with goat anti-mouse IgG conjugated with APC (Life technologies, Netherlands) 1:250 dilution at room temperature and mounted in ProLong^®^ Diamond Antifade Mountant with DAPI (Life technologies, United States). Non-infected cells and ZIKV-infected cells stained with mouse isotype IgG2a antibody (Dako, Denmark) were used as negative and isotype controls. Representative images of infected cells were generated on a Zeiss LSM 700 confocal laser scanning microscope fitted on an Axio Observer Z1 inverted microscope (Zeiss). All images acquired were processed using Zen 2010 software (Zeiss).

### Determination of Apoptotic Cells

Cells were grown on 96-well plate and infected at an MOI of 5. The number of apoptotic cells was measured using the terminal deoxynucleotidyl transferase dUTP nick end labeling (TUNEL) assay kit, *in situ* Cell Death Detection Kit, Fluorescein (Sigma Aldrich, United States). The cells were fixed with 4% PFA and permeabilized with triton X-100 1% and ethanol 70% (1:1 v/v). Uninfected cells were treated with 90 IU/ml DNAse (Roche, Germany) for 15 min to serve as a positive control (PC). TUNEL assay was performed based on the manufacturer’s instructions. Non-infected cells and ZIKV-infected cells stained only with labeling solution were used as negative controls (NCs) as suggested by the manufacturer. The number of TUNEL positive cells was measured using a BD FACS Canto II machine (BD Biosciences, United States). Data were analyzed using the FlowJo 10 software (FlowJo, United States).

### Thrombin Generation Time Test

Cells were grown on 96-well plate and infected with MOI of 0.1 and 5. Thrombin generation time (TGT) test was performed as previously described (Goeijenbier et al., 2015). Briefly, supernatant of cultured HUVECs on 96-well plate was discarded. Cells were washed three times with RPMI 1640 medium (Lonza, Netherlands) and 80 μL of pooled citrate plasma from healthy donors was added to the monolayer together with 60 μL HEPES buffer [25 mM Hepes, 137 mM NaCl, 0.1% Albumin]. As a standard, a serial dilution of recombinant TF (Innovin, Germany) was performed in the absence of cells. 60 μL HEPES calcium [25 mM Hepes, 137 mM NaCl, 0.1% Albumin,38 mM CaCl(2)] was then added and the plates were measured directly at optical density (OD) 450 nm using a Tecan Infinite 200 Pro ELISA reader (Tecan Group Ltd., Switzerland) in a kinetic cycle measuring every 45 s for 1 h. TGT was defined as the time at half the maximal OD. TF levels were also determined from the ELISA values as previously described (Goeijenbier et al., 2015). BPL-inactivated virus control was not used due to its interference with the assay.

### Quantification of Tissue Factor Expression on HUVECs

Human umbilical vein endothelial cells were analyzed for surface TF expression by flow cytometry. Briefly, HUVECs were grown on 96-well plate and infected at an MOI of 5. Infected cells were harvested, then stained with rabbit polyclonal anti-TF conjugated with Alexa Fluor 647 (Bioss Inc., United States) at a 1:200 dilution or isotype control in fluorescence activated cell sorter (FACS) buffer for 45 min on ice and in the dark. Subsequently, the cells were fixed and permeabilized with BD Cytofix/Cytoperm^TM^ solution (BD Biosciences, United States). Cells were incubated with 5% normal goat serum (DAKO, Denmark) in BD Perm/Wash solution (BD Biosciences, United States) for 10 min to minimize background. ZIKV was detected using mouse monoclonal antibody anti-flavivirus group antigen, MAB10216, clone D1-4G2-4-15 (Millipore, Germany) at a 1:400 dilution for 45 min on ice and in the dark. Subsequently, cells were incubated with goat anti-mouse IgG conjugated with Alexa Fluor 488 (Life technologies, Netherlands) at a 1:250 dilution for 45 min on ice and in the dark. Non-infected cells and ZIKV-infected cells stained with mouse isotype IgG2a antibody (DAKO, Denmark) were used as negative and isotype controls. The percentage of TF and infected cells were measured using a BD FACS Canto II machine (BD Biosciences, United States). Data were analyzed using the FlowJo 10 software (FlowJo, United States).

### IL-6 and IL-8 ELISA

Cells were grown on 96-well plate and infected at an MOI of 5. Supernatant of infected cells was collected and followed by centrifugation for 10 min at 930 *g*. IL-6 and IL-8 levels from the supernatant were measured using IL-6 (Quantikine R&D, United States) and IL-8 (Quantikine R&D, United States) ELISA kits according to the manufacturer’s instructions.

### IL-6 and IL-8 Blocking Experiments

Cells were grown on 96-well plate and infected at an MOI of 5. Following normal infection experiment as the above protocol, anti-IL-6 (Quantikine R&D, United States) or IL-8 antibody (Quantikine R&D, United States) was added to the wells according to our ELISA results and the manufacturer’s recommendation. TF expression was subsequently determined with flow cytometry at 24 and 48 hpi as the above protocol. To confirm that the blocking experiments work, we incubated HUVECs with IL-6 and IL-8 concentrations that we observed in our study and determined the downstream activation pathway of these cytokines. Briefly, HUVECs were grown on 96-well plate and was cultured for 24 or 48 h. Dilution of anti-IL-6 or -IL-8 antibody was mixed with either IL-6 or IL-8 protein (Quantikine R&D, United States) based on the concentrations that we observed in our study for 30 min at 37°C before it was added to the cells. The cells were then cultured for 4 h. Next, RNA was isolated from HUVECs with High Pure RNA Isolation kit (Roche, Germany). Subsequently, cDNA synthesis was performed with Superscript IV (Thermo Fisher Scientific, United States). Expression of nuclear factor kappa B subunit 1 (NFKB1), tissue inhibitors of metalloproteinases-1 (TIMP1) and hypoxia-inducible factor 1-alpha (HIF1A) genes were determined with real-time PCR using the Taqman Universal PCR Master mix II according to the manufacturer’s recommendation (Applied Biosystems, United States). Gene expression was corrected for the housekeeping gene glyceraldehyde 3-phospate dehydrogenase (GAPDH) as described previously (Schmittgen and Livak, 2008). All the primer-probe assays were obtained from Applied Biosystems, United States.

### Statistical Analysis

The statistical analyses were performed using GraphPad Prism 5.01 software for Windows. Student’s *t*-test or Mann Whitney *U* test was used for the comparison of mean and median, respectively, between two groups. For comparison between multiple groups, one-way analysis of variance (ANOVA) test with Tukey’s multiple comparison or Kruskal-Wallis test with Dunn’s multiple comparison was used. *P* values ≤ 0.05 were considered significant.

## Results

### ZIKV Infects and Replicates in Primary Human Umbilical Vein Endothelial Cells

**FIGURE 1:**
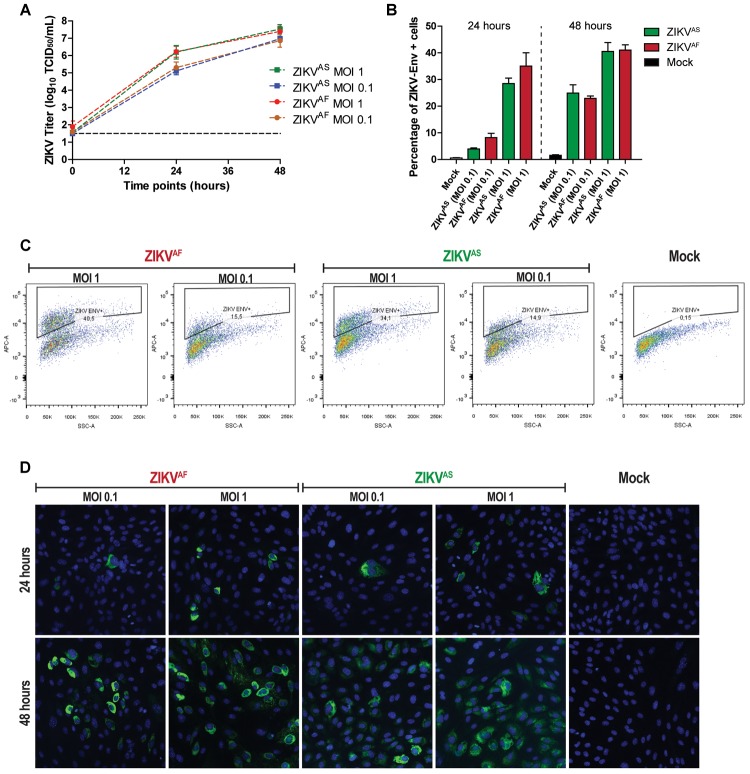
Zika virus (ZIKV) infects and replicates efficiently in human umbilical vein endothelial cells (HUVECs). HUVECs were infected with two ZIKV strains at two multiplicity of infections (MOIs; 1 and 0.1) and samples were collected at the designated times. **(A)** Infectious titers of supernatants collected at 0, 24, and 48 h post-infection (hpi). Experiments were done in triplicate and data are representative of three independent experiments. Bars represent standard error of the mean (SEM). **(B)** HUVECs were infected at two MOIs (0.1 and 1) and stained for the presence of ZIKV envelope by flow cytometry at 24 and 48 hpi. Experiments were done in triplicate and data are representative of two independent experiments. The results are displayed as mean and bars represent SEM. **(C)** Representative flow cytometry analyses plot to determine the percentage of infected cells at 48 hpi. **(D)** HUVECs were infected with two ZIKV strains and then imaged for the presence of viral envelope protein at 24 and 48 hpi. 200× magnification.

To confirm that ZIKV infects and replicate in ECs, we determined the replication kinetics of two strains of ZIKV, one of African lineage and one of Asian lineage, using low MOIs (0.1 and 1). In accordance with the previous study, (Liu et al., 2016) we found that HUVECs supported infection for both ZIKV^AF^ and ZIKV^AS^. Both virus strains replicated to a titer of 10^7^ TCID_50_/ml without noteworthy differences in the replication kinetics (Figure 1A). These results were supported by flow cytometry and IFA staining. The flow cytometry data showed that at both MOI of 0.1 and 1 the number of infected cells increased on average 5–10% (MOI 0.1) and 27–35% (MOI 1) at 24 hpi to ±25% (MOI 0.1) and ±40% (MOI 1) at 48 hpi (Figure 1B). The data shown in Figure 1C are representative data of the flow cytometry analyses to determine the percentage of infection. Flow cytometry results were also confirmed by IFA intracellular staining of ZIKV infected cells at low MOIs (Figure 1D). Taken together, these data confirm that ZIKV could infect and replicate efficiently in HUVECs.

### Increased TF Concentrations and Shortened Thrombin Generation Time After ZIKV Infection of HUVECs

**FIGURE 2:**
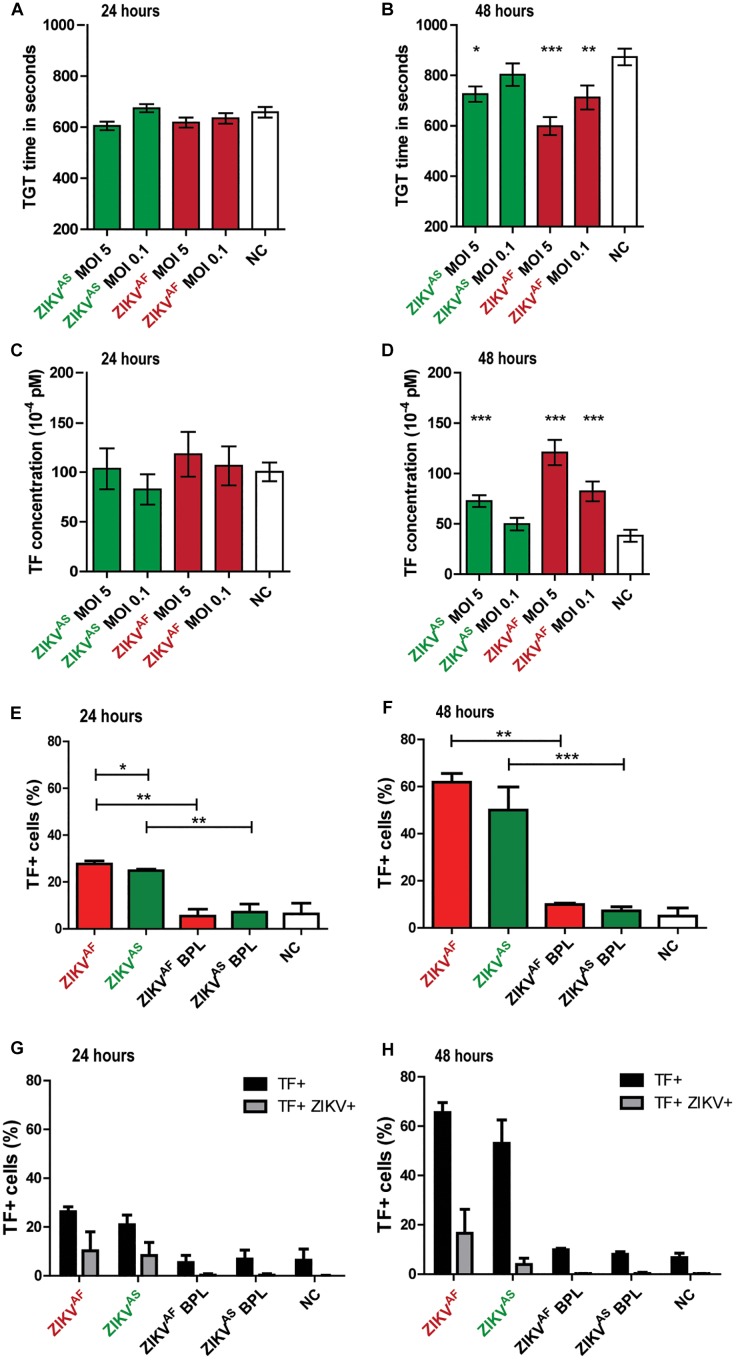
Zika virus infection of HUVECs leads to increased tissue factor (TF) expression and activation of secondary hemostasis. HUVECs were infected with an MOI of 0.1 or 5 with both ZIKV strains and thrombin generation time (TGT) test was performed as a functional assay to assess secondary hemostasis at 24 and 48 h post-infection **(A,B)**. TGT was significantly shortened for ECs infected with ZIKV^AF^ (MOI 0.1 and 5) and ZIKV^AS^ (MOI 5) at 48 hpi **(B)**. **(C,D)** TF concentration was calculated from a standard curve to determine whether there is an increase of TF expression at 24 and 48 hpi based on previous study (Goeijenbier et al., 2015) TF expression increased significantly at 48 hpi for both strains (ZIKV^AF^: MOI 0.1 and 5; ZIKV^AS^: MOI 5). Experiments were done in eight replicates and data are representative of three independent experiments. **(E,F)** TF expression was also confirmed with flow cytometry. Increased TF expression was observed for both virus strains against BPL and negative control (NC) at 24 and 48 hpi. Approximately 61 % of cells expressed TF at 48 hpi for ZIKV^AF^. **(G,H)** Double staining experiments were performed to determine whether increased TF expression occurred in infected or bystander cells. Black bars represent the total amount of TF+ cells while gray bars represent the percentage of double positive cells for both ZIKV and TF. Less than 40% of total TF+ cells were double positive for both ZIKV and TF at 24 hpi **(G)**. Meanwhile, <25% of total TF+ cells were double positive for both ZIKV and TF at 48 hpi **(H)**. Experiments were done in triplicate and data are representative of two independent experiments. All the results are displayed as mean and bars represent SEM. Statistical analyses were performed against mock. Statistical significance is shown: ^∗^*p* < 0.05; ^∗∗^*p* < 0.01; ^∗∗∗^*p* < 0.001.

To test whether ZIKV infection induces activation of secondary hemostasis, we incubated HUVECs with both ZIKV strains at a low MOI (0.1) and high MOI (5) or virus-free cultured medium from Vero cells. Two ZIKV lineages were used to assess whether activation of secondary hemostasis is lineage-specific or not. We measured TGT as a functional assay to assess secondary hemostasis. Thrombin generation was quantified directly on infected cells by incubating plasma on cells and initiating coagulation by the addition of calcium ions (Figure 2A,B). We observed that ZIKV-infected HUVECs triggered plasma clotting faster compared than mock control (Figure 2B). The significant increase was mainly seen at 48 hpi for both strains (*p* < 0.0001 MOI 5 ZIKV^AF^, *p* = 0.0007 MOI 1 ZIKV^AF^; *p* = 0.0002 ZIKV^AS^). We further quantified the mean TF concentrations after ZIKV infection, calculated from TGT standard curve as described previously (Figure 2C,D) (Goeijenbier et al., 2015). TF concentrations were significantly increased for ZIKV^AF^ at both MOIs at 48 hpi (*p* = 0.0007 MOI 0.01; *p* < 0.0001 MOI 5) and only at MOI 5 for ZIKV^AS^ (*p* = 0.0002) (Figure 2D). Since we only observed significant differences mainly with MOI of 5 and based on the *in vitro* studies of DENV (Dewi et al., 2004) and puumala virus (Goeijenbier et al., 2015), which showed that a high MOI is needed to observed permeability and/or hemostatic disorders *in vitro* (HUVECs models), we decided to continue with this MOI for the rest of the experiments. We noticed that increased TF expression were detected since 24 hpi (Figure 2E) and was higher at 48 hpi (Figure 2F). Both ZIKV strains significantly increased TF concentrations compared to BPL at 24 hpi (ZIKV^AF^: *p* = 0.0022; ZIKV^AS^: *p* = 0.002) and 48 (ZIKV^AF^: *p* = 0.002; ZIKV^AS^: *p* = 0.0004).

To determine whether increase TF levels occurred in infected or bystander cells, we performed double staining assays against TF and ZIKV envelope (Figure 2G,H). We measured TF on both ENV+ and ENV- cells, but increased TF mainly occurred in the bystander (ENV-) evidenced by the fact that <26% of all the positive TF cells were double positive cells at 48 hpi (ZIKV^AF^: 25.5%; ZIKV^AS^: 7.5%) (Figure 2H). Collectively, our data indicate that ZIKV infection on HUVECs induces increased TF concentration mostly through bystander effect that allows activation of secondary hemostasis.

### ZIKV Infection of HUVECs Induces Apoptosis Mainly Through Infection

**FIGURE 3:**
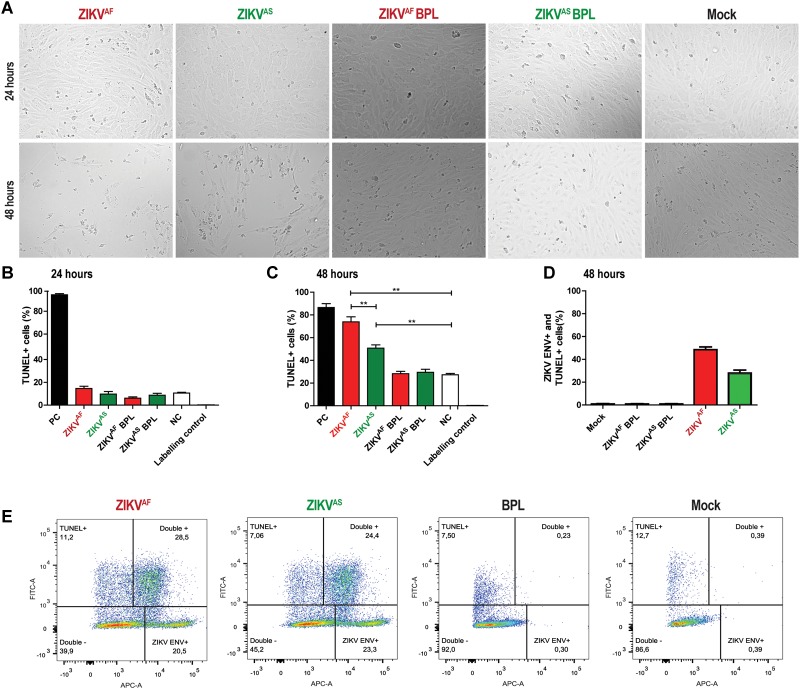
Zika virus infection on HUVECs induces cell death mainly through infection. HUVECs were infected with a MOI of 5 with both ZIKV strains. **(A)** Cytopathic effect (CPE) was observed at 48 h post-infection (hpi) for both ZIKV strains with light microscopy at 200× magnification. More CPE was observed with ZIKV^AF^. **(B,C)** To measure cell death, cells were stained for DNA fragmentation with the terminal deoxynucleotidyl transferase dUTP nick end labeling (TUNEL) assay kit at 24 and 48 h post-infection (hpi). Both ZIKV strains induced cell death at 48 hpi. ZIKV^AF^ induced more cell death compared to ZIKV^AS^ (*p* < 0.01). Experiments were done in triplicate and data are representative of two independent experiments. **(D)** To determine whether cell death was caused by direct infection or bystander effect, we performed double staining directed against ZIKV envelope protein and TUNEL with flow cytometry. HUVECs were infected at an MOI of 5 and samples were collected at 48 hpi. Majority of the TUNEL+ cells were also positive for ZIKV ENV. **(E)** Representative flow cytometry analyses plot to determine the ZIKV ENV+ and TUNEL+ cells at 48 hpi. Experiments were done in triplicate and data are representative of two independent experiments. All the results are displayed as mean and bars represent SEM. Statistical significance is shown: ^∗^*p* < 0.05; ^∗∗^*p* < 0.01; ^∗∗∗^*p* < 0.001.

Previous studies indicated that cell apoptosis could activate the coagulation system (Greeno et al., 1996; Yang et al., 2017). In addition, several studies showed that ZIKV infection of several primary cells induced cell death (Quicke et al., 2016; Anfasa et al., 2017). To determine whether ZIKV infection induced cell death of HUVECs, we assessed cell viability using a TUNEL assay. We found that infection with both strains led to significant morphological changes and increased cell death at 48 hpi (Figure 3A). No significant differences in cell death were observed at 24 hpi between the groups (Figure 3B). Cells treated with DNAse showed approximately 90% of TUNEL+ cells at both 24 and 48 hpi. ZIKV^AF^ induced significantly more cell death (*p* = 0.005) than ZIKV^AS^ at 48 hpi (65% vs. 55%) (Figure 3C). To determine whether cell death occurred in infected or bystander cells, we performed double staining experiments against ZIKV envelope and TUNEL positive cells. We found that cell death mainly occurred due to infection, especially with ZIKV^AF^ (Figure 3D,E). Approximately 60% of the total death cells were also positive for ZIKV^AF^ while the number was 40% for ZIKV^*AS*^. Collectively, our data indicate that ZIKV infection of HUVECs leads to increased cell death mainly in the infected cells.

### ZIKV Infection on HUVECs Triggers Production of Pro-inflammatory Cytokines

**FIGURE 4:**
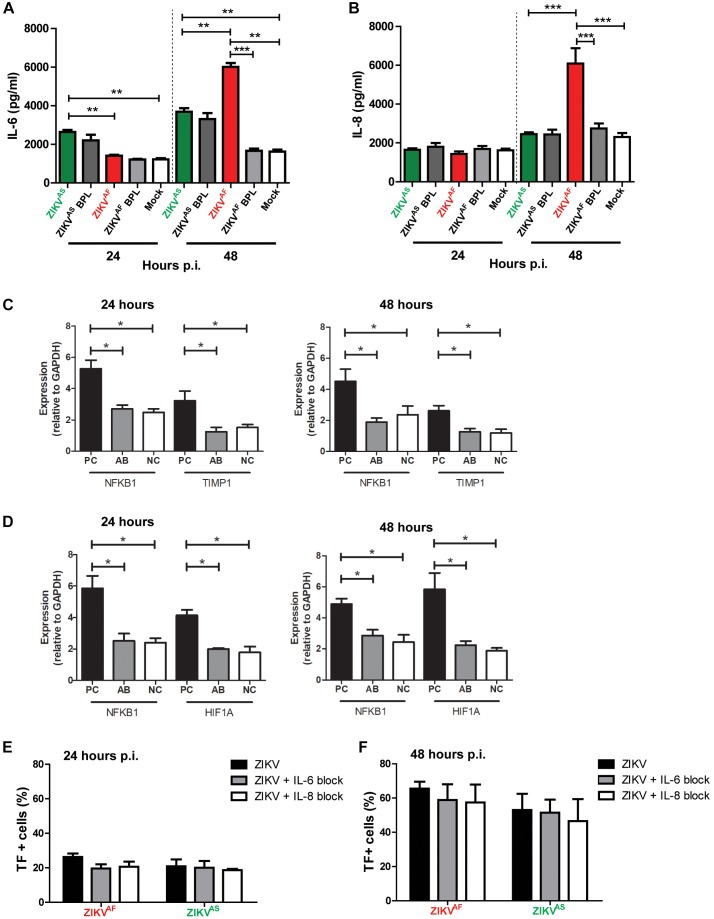
Zika virus-infected HUVECs produce pro-inflammatory cytokines. HUVECs were infected with a MOI of 5 with both ZIKV strains and supernatants were collected at the designated times. Significantly, increased levels of IL-6 **(A)** and IL-8 **(B)** were detected at 48 h post-infection (hpi) for ZIKV^AF^. **(C,D)** To investigate whether the concentrations of anti-IL-6 and -IL-8 that we used were sufficient to neutralize IL-6 and IL-8 concentrations at 24 and 48 hpi, we determined the gene expression levels of NFKB1, TIMP-1, and HIF1A, which are the downstream activation pathways of IL-6 (NFKB1 and TIMP1) and IL-8 (NFKB1 and HIF1A). The gene expression levels of NFKB1 and TIMP1 were significantly lower in IL-6 with anti-IL-6 treatment (AB) compared to positive controls (PC; IL-6 protein only) and similar to negative controls (NCs) **(C)**. The gene expression levels of NFKB1 and HIF1A were significantly reduced in IL-8 with anti-IL-8 treatment **(A,B)** compared to PC (IL-8 protein only) and equal to NC **(D)**. **(E,F)** Modest TF expression reduction was observed after IL-6 and IL-8 blocking experiments at 24 and 48 hpi. The results are based on three (Panels **(A,B)** and two (Panels **C–F)** independent experiments. All experiments were performed in triplicate except for Panels **(C,D)** that was performed in duplicate. The results are displayed as mean and bars represent SEM. Statistical significance is shown: ^∗^*p* < 0.05; ^∗∗^*p* < 0.01; ^∗∗∗^*p* < 0.001.

Previous studies suggested that IL-6 and IL-8 can also increase TF expression on HUVECs and monocytes (Wada et al., 1995; Neumann et al., 1997). To assess whether ZIKV infection led to increased production of IL-6 and IL-8, the culture supernatant of infected HUVECs was collected at 0, 24, and 48 hpi. We found that IL-6 and IL-8 concentrations were significantly increased in the supernatant of ZIKV infected-cells compared to the controls. ZIKV^*AF*^ induced a higher production of IL-6 and IL-8 than ZIKV^AS^, BPL-inactivated virus and mock control at 48 hpi (Figure 4A). IL-6 and IL-8 levels increased significantly compared to BPL-inactivated virus (IL-6: *p* = 0.0018; IL-8: *p* < 0.0001) and NC (IL-6: *p* = 0.0001; IL-8: *p* < 0.0001) 2 days after infection. ZIKV^AS^ induced increased levels of IL-6 at 24 (*p* < 0.0001) and 48 (*p* < 0.0001) hpi compared to mock control (Figure 4A,B). Interestingly, ZIKV^AS^ BPL control also induced elevated levels of IL-6 production.

To determine whether increase IL-6 and IL-8 production was linked to increased TF productions, we further performed blocking experiments against both cytokines and measured TF levels at 24 and 48 hpi. We first investigated whether the concentrations of anti-IL-6 and -IL-8 that we used could neutralize IL-6 and IL-8 by determining the expression levels of several genes of the downstream activation pathway of both cytokines. We found that the gene expression levels of NFKB1 and TIMP1 were significantly reduced in IL-6 with anti-IL-6 treatment (AB), comparable to (NC; medium only), compared to cells with IL-6 only (PC) at both 24 and 48 hpi (Figure 4C). Similarly, NFKB1 and HIF1A expression levels were reduced in the presence of anti-IL-8 (Figure 4D). These results suggest that both monoclonal antibodies could neutralize the respective cytokines. Next, blocking experiments were conducted and TF expression was measured. The results revealed modest reduction of TF (Figure 4E,F), which suggest that IL-6 and IL-8 did not contribute significantly to the increased TF expression on HUVECs.

## Discussion

The present study addresses the effect of ZIKV infection of ECs *in vitro* on TF expression and apoptosis. We observed that infection with both an African and an Asian strain of ZIKV resulted in increased TF expression on HUVECs, which led to a shortened TGT. We showed that increased TF expression were mainly induced in ENV-positive cells, which indicate an indirect effect of infection on TF expression. It is important to note that very early stage of infection may lead to increased TF expression where the levels of ENV expression is still under the limit of detection.

Tissue factor is a major activator of the coagulation cascade during virus infections (Antoniak and Mackman, 2014). A previous study with EBOV indicated that the over-expression of TF in primate monocytes/macrophages plays an important role in triggering the pro-coagulant state and consequently hemorrhagic complications (Geisbert et al., 2003). The role of TF in hemorrhagic fevers has also been described in dengue in that infection of ECs with DENV resulted in increased TF expression via the phosphorylation of p38 and ERK1/2 MAPKs (Huerta-Zepeda et al., 2008). Excess TF production during infection could thus lead to increased clotting and eventually consumptive coagulopathy or even disseminated intravascular coagulation (DIC). Our results support the recent observations in humans and animal models, which suggest that hemostasis alterations occur during ZIKV infection (Brasil et al., 2016a,b; Chraibi et al., 2016; Karimi et al., 2016; Miner et al., 2016; Sarmiento-Ospina et al., 2016; Sharp et al., 2016; Zonneveld et al., 2016; Nguyen et al., 2017; Wu et al., 2017).

Liu et al. (2016) showed recently that ZIKV could infect ECs from different vascular beds. Interestingly, the authors found that HUVECs were the most susceptible to ZIKV infection compared to human ECs derived from aortic and coronary artery, as well as the saphenous vein. Moreover, a recent study found that ZIKV NS1 specifically induced vascular leakage in HUVECs and brain ECs from several primary ECs that were tested (Puerta-Guardo et al., 2019). EC heterogeneity has been described between different organs, both on molecular, and functional levels (Aird, 2012). Therefore, infection of ECs from different vascular beds with certain pathogens may lead to different pathogenic outcomes. This hypothesis has been supported for other hemorrhagic viruses such as DENV and puumala virus (Peyrefitte et al., 2006; Goeijenbier et al., 2015). In contrast to our results, *in vitro* studies using human brain microvascular endothelial cells (hBMECs) and human retinal microvascular ECs observed limited or no cytopathology during ZIKV infection (Mladinich et al., 2017; Roach and Alcendor, 2017). Moreover, Mladinich et al. (2017) found that ZIKV infected and persisted in hBMECs. Whether vascular heterogeneity play a role in the pathogenesis of ZIKV-induced brain disorders and hemostasis alterations warrants further study.

It is known that pro-inflammatory cytokines play an important role in activation and downregulation of the coagulation system. We found that ZIKV infection of HUVECs leads to increased levels of IL-6 and IL-8 production. Our data is in line with a study that showed increased IL-6 production of ZIKV-infected retinal ECs (Roach and Alcendor, 2017). We observed elevated levels of IL-6 also in HUVEC incubated with BPL-inactivated ZIKV^AS^ suggesting that binding of viral proteins to EC alone may stimulate production of IL-6. This is in line with the study of Roach and Alcendor (2017) that used a heat-inactivated Asian strain (PRVABC59) of ZIKV and reported increased levels of IL-6 production compared to mock control. We further blocked IL-6 and IL-8 during infection to determine whether these cytokines contributed to increased TF expression using concentrations that were showed to neutralized these cytokines (Takeuchi et al., 2015). Our results indicated a modest TF expression reduction when both IL-6 or IL-8 were blocked, indicating limited effect of these cytokines neutralization on TF expression.

Several reports revealed that ZIKV infection of primary cells induced apoptosis (Tang et al., 2016; Anfasa et al., 2017). In the present study, we observed that both ZIKV strains induced significant cell death. It is noteworthy that approximately 20% of dead cells were found in the mock control after 48 h of culture, which might be explained by the donor since we observed differences among various donors (data not showed). It has been shown that apoptotic cells are procoagulant (Bombeli et al., 1997; Bouvy et al., 2014; Yang et al., 2017). Although the mechanism of how apoptotic cells activate coagulation system remains to be determined, it was shown that both adherent and detached apoptotic HUVECs become procoagulant by increased expression of phosphatidylserine (PS) and the loss of anticoagulant components such as TM and TFPI (Bombeli et al., 1997). Another possibility is that apoptotic ECs produce extracellular vesicles contributing to a procoagulant phenotype of the cells (Bouvy et al., 2014).

Our study has several limitations. First, we did not determine the whole spectrum of the hemostasis pathway. Second, we did not address the other factors that could trigger TF expression on HUVECs. Therefore, further studies to understand the mechanism of hemostasis activation during ZIKV infection is needed. However, our study provides evidence of increased TF expression, mostly due to bystander effect, that leads to activation of secondary hemostasis.

## Author Contributions

FA, MG, JM, and BM conceptualized and designed the study. FA, MG, WW, JS., NM, NO, and DR performed the experiments. FA, MG, WW, MK, BR, JM, and BM analyzed the data. FA and BM wrote the manuscript. All authors read and approved the final version of the manuscript.

## Conflict of Interest Statement

The authors declare that the research was conducted in the absence of any commercial or financial relationships that could be construed as a potential conflict of interest.
